# Evaluation of Xpert Carba-R Assay for the Detection of Carbapenemase Genes in Gram-Negative Bacteria

**DOI:** 10.1155/2021/6614812

**Published:** 2021-04-08

**Authors:** Hou-He Li, Zhi-Jian He, Li-Min Xie, Jin-Sheng Zhang, Tian-Ao Xie, Shu-Jin Fan, Xu-Guang Guo

**Affiliations:** ^1^Department of Clinical Laboratory Medicine, The Third Affiliated Hospital of Guangzhou Medical University, Guangzhou 510150, China; ^2^Department of Clinical Medicine, The Third Clinical School of Guangzhou Medical University, Guangzhou 511436, China; ^3^Key Laboratory for Major Obstetric Diseases of Guangdong Province, The Third Clinical School of Guangzhou Medical University, Guangzhou 510150, China; ^4^Key Laboratory of Reproduction and Genetics of Guangdong Higher Education Institutes, The Third Clinical School of Guangzhou Medical University, Guangzhou 510150, China; ^5^Stomatological Hospital of Guangzhou Medical University, 510150, China

## Abstract

**Introduction:**

High mortality associated with carbapenemase-producing Gram-negative bacteria (CP-GNB) has evolved into a global health threat. Rapid and accurate detection as well as prompt treatment are of great significance in this case. Xpert Carba-R, a multiple qualitative analysis designed to detect five clinically relevant carbapenem-resistant gene families within one hour, is regarded as reliable, accurate, and easy-to-operate. This study is to present a systematic evaluation of the performance of Xpert Carba-R in detecting carbapenemase genes in GNB suspected for carbapenemase production.

**Methods:**

We searched and screened the literature on “Xpert Carba-R” in the database of PubMed, Web of Science, Embase, and Cochrane Library, employing two independent evaluators to collect data, respectively. Then, statistical analysis of the data obtained was performed by the Stata 12.0 software to measure the accuracy of Xpert Carba-R assay in detecting the carbapenemase genes in GNB.

**Results:**

We screened a total of 1767 Gram-negative bacillus isolates documented in 9 articles. The precision of the detection of OXA-48 carbapenemase genes was 100%; that of NDM = 100%; that of VIM = 100%. When it came to KPC, the precision rate was 100%; that of IMP = 99%. The overall accuracy of the detection of carbapenemase genes was 100%.

**Conclusions:**

Xpert Carba-R assay demonstrates a 100% precision in identifying carbapenemase genes in GNB. It can be seen that Xpert Carba-R method is an effective tool for early clinical detection, which is suitable for the detection of carbapenase gene in GNB.

## 1. Introduction

The main mechanism of carbapenem resistance of Gram-negative bacteria (GNB) is the production of carbapenemase, which hydrolyzes many types of antibiotics (carbapenems, cephalosporins, penicillins, and aztreonam) [1, 2]. Early in 2005, Walsh et al. specifically pointed out that the continuous spread of carbapenem-resistant Gram-negative bacteria (CR-GNB) worldwide will lead to clinical disasters and perhaps future public health crises [3]. Among them, carbapenem-resistant Enterobacteriaceae (CRE) especially has stand out from the crowd in that they are resistant to a great variety of drugs. Report had it that CRE caused 9,000 infections and 600 deaths each year in the United States [4, 5]. Currently, the dramatic increase in the prevalence and clinical impact of infections caused by carbapenemase-producing Gram-negative bacteria (CP-GNB) has snowballed into a global health concern for the invasive infection of these bacteria is inextricably associated with a high mortality rate [1, 6].

Currently, phenotypic methods are often used to detect carbapenemase resistance in Gram-negative bacteria in clinical laboratories, and to explore the molecular basis of carbapenemase resistance [7, 8]. The modified Hodge test (mHT), as a good case in point, is still frequently employed in detecting carbapenem product of GNB, which is, however, condemned as not desirable for disease control and treatment as it is time-consuming and severely limited by both the specificity and sensitivity in analysis.

Xpert Carba-R assay, a PCR-based test run on the GeneXpert platform, is designed for the rapid detection and differentiation of 5 carbapenemase genes (blaKPC, blaIMP, blaNDM, blaVIM, and blaOXA-48-like) [9]. The operation of Xpert Carba-R assay requires only 2 simple steps that could be completed within 1 hour, with less than 1 minute of hands-on time. As instructed by the manufacturer, researchers add 10 *μ*L solution of 0.5 L McF standard suspension of the sample to a 5 mL Xpert Carba-R sample reagent vial and mix for 10 seconds. Through the supplied pipette, the sample reagents were added to the Xpert Carba-R kit and analyzed by the Cepheid GeneXpert platform [10, 11]. The result proves Xpert Carba-R analysis a reliable, accurate, and easy-to-use multiple qualitative analytic tool that is able to detect five clinically relevant carbapenemase gene families directly from the rectal swabs, which takes reduced time comparing with other culture-based methods for the identification of patients with gastrointestinal colonization of carbapenem-resistant organisms (CPO) [12, 13]. The purpose of this study is to evaluate the performance of Xpert Carba-R assay in detecting carbapenemase genes in GNB, thereby providing a good epidemiological tool and a reference standard for clinical diagnosis.

## 2. Materials and Methods

### 2.1. Study Design and Literature Review Strategy

The research was scheduled from October 2019 to the present time, designed to be a systematic evaluation of the accuracy of Xpert Carba-R assay in the identification of carbapenemase genes.

We searched four databases, namely, PubMed, Web of Science, Embase, and Cochrane Library, with the keywords “Xpert Carba-R assay.” Articles published before October 2019 were collected and imported into the EndNoteX9 software for file management. At the same time, we have also formulated the corresponding inclusion and exclusion criteria (Table 1).

### 2.2. Literature Screening and Data Extraction

All literature was screened according to the exclusion and inclusion criteria established previously, and a PRISMA flowchart was formed. The EndNoteX9 software was employed only for document management, and the information of each published study, including the author, year of publication, region, and other related information, was also extracted and included into a table for presentation.

### 2.3. Quality Evaluation of Literature

QUADAS-2 [13] worked as the instrument for quality appraisal, which was evaluated by two raters independently. Should the result reveals any inconsistency, it would subject to the discussion with a third person in the group.

### 2.4. Statistical Analysis

The main index of the performance of Xpert Carba-R assay is the accuracy in the determination of carbapenemase genes in GNB, which would be presented by the statistics processed by the Stata 12.0 software. Moreover, the *I*^2^ value reveals the heterogeneity of researches in a way that a greater *I*^2^ value indicates a higher heterogeneity. Should the heterogeneity be great enough, an additional subgroup analysis of the previously included studies would be performed to investigate the amount and extent of the effect that the suspected factors contribute to the heterogeneity. The data adopted to examine the levels of GNB from each study would generate an impact on the aggregate effect as a whole so that we did an impact analysis. The included studies were evaluated for publication bias with the Egger's test.

## 3. Result

### 3.1. Data Screening and Inclusion

We had a thorough search over the database of PubMed, Web of Science, Embase, and Cochrane Library with words, terms, and sentences related to this study. 44 articles were selected out from PubMed, 47 from Web of Science, 72 from Embase, and none from Cochrane Library, i.e., 163 articles in total. No related articles were retrieved. Apart from 80 articles excluded for duplication, the remaining 83 articles were subject to a full-text screening, by means of which another parts of articles were excluded, including those that did not meet the gold standard, those that data could not be extracted from, and those unrelated to our research. Nine articles were finally included in our research, and a systematic analysis of them was conducted [9, 10, 14–20].

Data of the performance of Xpert® Carba-R in detecting various types of carbapenemase genes were extracted from the 9 articles included and were summarized in Table 2, which demonstrated a high accuracy of Xpert Carba-R in the detection of carbapenemase genes.

### 3.2. Systematic Analysis

**Figure 1:**
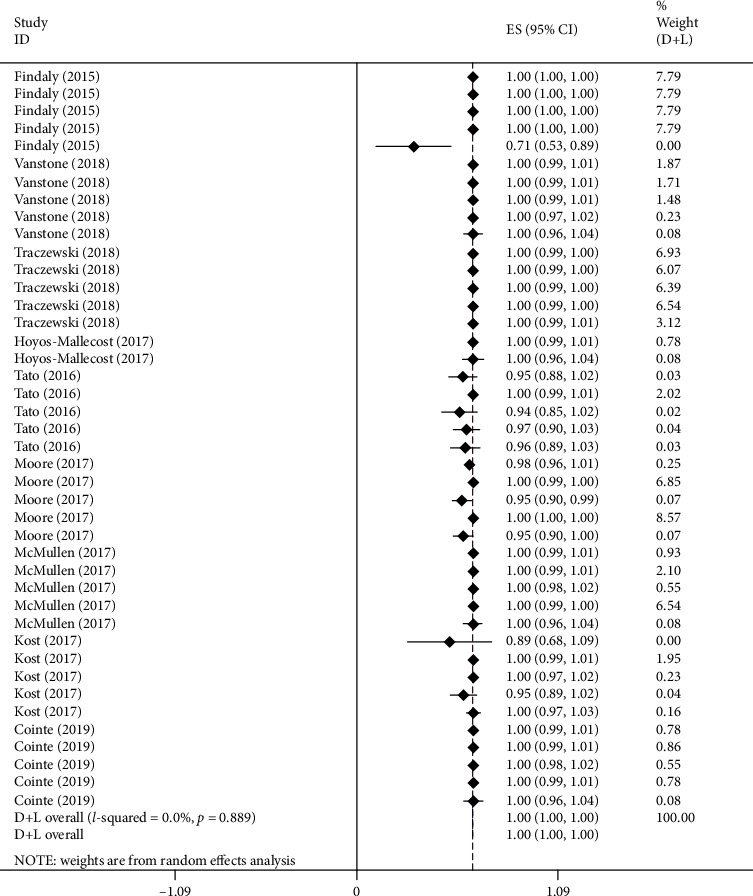
Forest map for the analysis of the carbapenemase gene Identification ratio at the genetic level.

A total of 1767 Gram-negative bacilli in 9 articles were evaluated (Table 3), and the Stata software was employed to conduct a systematic review of the 9 articles, which was displayed in the form of a forest map (S1). At the same time, we also summarized the data characteristics of the included studies and presented them in Table 4. The precision of the detection of OXA-48 carbapenemase gene was 100%, with a *p* value of 0.753 and an *I*^2^ value of 0.0%; that of NDM was 100%, with a *p* value of 1.000 and an *I*^2^ value of 0.0%; that of VIM was 100%, with a *p* value of 0.403 and an *I*^2^ value of 3.5%; that of KPC was 100%, with the *p* value being 0.931 and the *I*^2^ value 0.0%; that of IMP was 99%, with the *p* value being 0.039 and the *I*^2^ value 52.6%. The overall accuracy of the detection of carbapenemase genes was 100%, with a *p* value of 0.889 and an *I*^2^ value of 0.0%. The results are exhibited in Figure 1.

### 3.3. Sensitivity Analysis

**Figure 2:**
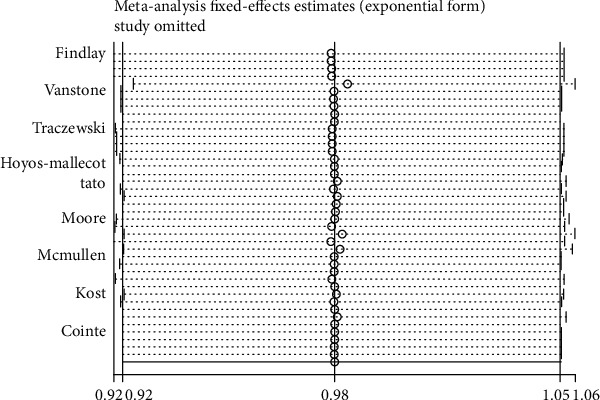
Sensitivity analysis of carbapenemase genes.

Sensitivity analysis is designed to assess the impact of every one of the nine studies on the consolidated results of the systematic analysis by comparing the amount of the combined effect generated by the analysis excluding one of the 9 studies and that produced by the analysis of all studies included. On the left side, the vertical line represents the minimum value of the 95% confidence interval for the overall consolidation effect, and the vertical solid line on the right represents the maximum value for the 95% confidence interval for the overall consolidation effect. The overall effect is represented by a solid vertical middle line of 0.98 (0.92 and 1.05). The results are shown in Figure 2.

### 3.4. Publication Bias

**Figure 3:**
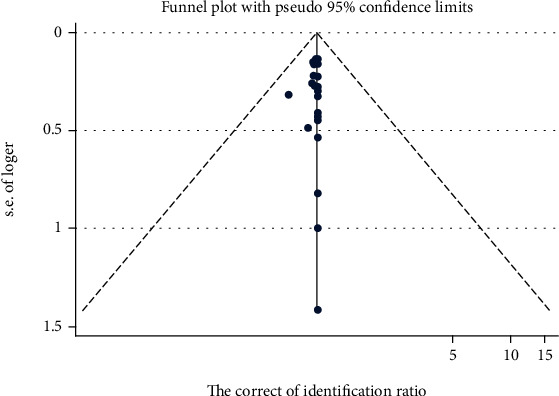
Funnel plot incorporating carbapenemase genes.

As a matter of fact, there is a tendency to publish or report studies with results that support a hypothesis than those that do not so that the negative information obtained from the database may be unscientifically modulated, thereby causing systematic error, which is named publication bias. In order to have a good grip of the impact of publication bias on the literature review of this research, all data extracted from the articles included were processed into a funnel chart. The results of the combination of carbapenemase genes are displayed in Figure 3, with a *t* value of -0.35 and a *p* value of 0.728.

## 4. Discussion

Since a national outbreak of a disease caused by carbapenem-resistant Enterobacter (CRE) appeared in Israel in 2006 [20], how to address GNB's resistance to carbapenem antibiotics has become a heated topic in public health [21]. Rapid detection of CR-GNB infection or carbapenem resistance is of great significance to reduce the mortality rate [22]. Xpert Carba-R assay, as an emerging real-time PCR-based detection instrument, boasts short turnaround time (<1 hour), simple and fast operation, high detection rate, and low cost [23]. Compared with the traditional PCR method that is still widely accepted as the gold standard, Xpert Carba-R assay is an important improvement in the diagnosis of diseases caused by CR-GNB.

The main purpose of this study was to demonstrate the performance of Xpert Carba-R assay for the determination of carbapenemase genes in GNB. Included in the study were the detection rates obtained by testing samples that had been previously confirmed by the gold standard. After strict screening based on the formerly established inclusion and exclusion criteria, 9 articles were finally selected and data extracted from them were processed and analyzed with the designated statistical software. The final results show that Xpert Carba-R assay performs a 100% (*p* = 0.889 and *I*^2^ = 0.0%) accuracy, which justifies that it is a well-suited method for the detection of carbapenemase genes. In this study, the “big five” families of carbapenemase genes, namely, OXA-48, KPC, IMP, NDM, and VIM were investigated. In the process of literature review, Xpert Carba-R assay turned out to detect two carbapenemase genes in the same sample simultaneously, such as OXA-48-NDM, OXA-48-KPC [24], KPC-2-VIM-1, and IMP-1-VIM-1 [25]. However, due to strong randomness of these cases and for the accuracy of scientific research, there will be no further elaboration on that in this article. What is more, Xpert Carba-R assay is highly inclusive in terms of the source of samples, by which the authors mean that various types of clinical specimens could serve as the samples, including urine, blood, body fluid, respiratory tract sampling., and rectal swab [10]. In Hoyos-Mallecot et al.'s study, in view of diagnostic accuracy, Xpert Carba-R assay behaves even better than the gold standard [15]. However, for the sake of the rigorousness of statistical analysis, data were still extracted based on the results of gold standard test.

From another perspective, Xpert Carba-R assay has certain limitations. For instance, Xpert Carba-R assay could not identify some subtypes of genes. To name a case in point, in Findlay et al.'s study, Carba-R assay cannot identify OXA-181, one of the subtypes of the OXA-48 family [14], and it is the same case with OXA-232 [9]. This may be a result of the imperfections of the technology corresponding to the database. Another limitation of Xpert Carba-R assay lies in that although Xpert Carba-R assay could effectively detect various types of samples, the samples must be cultured in advance [9]. At the same time, the decrease of the positive rate may also be due to the low bacterial load of the samples [12]. Moreover, different types of samples are always accompanied with varied sensitivities of the tests [21], which are the problems that await more advanced Xpert Carba-R assay to solve.

A subgroup analysis of the “big five” family of carbapenemase genes was performed to explore the heterogeneity of the studies. The detection rates of OXA-48, NDM, and KPC are all 100% (*I*^2^ = 0.0%), which was mentioned in the previous part of this article, indicating that there is no heterogeneity. In the VIM and IMP families, the detection rate of the former is 100% (*I*^2^ = 3.5%), signifying a low heterogeneity that can be ignored; the detection rate of the latter is 99% (*I*^2^ = 52.6%), 50% < 52.6% < 75%, revealing moderate heterogeneity in this subgroup. The overall rate of carbapenemase gene detection is 100% (*p* = 0.886 and *I*^2^ = 0.0%), which indicates that the source of heterogeneity in this study was not a grouping factor. By analyzing the source literature where the data of the IMP subgroup coming from, it is speculated that the source of heterogeneity of this subgroup may be related to different gold standards for the selection of studies, a lack of continuity in the adoption of strain samples, variety of the types of samples, and some human factors [9–11, 14–20]. For example, the IMP samples in Findlay et al.'s study were obtained randomly based on geographical factors, which may lead to heterogeneity within the subgroup [14].

The analytic results of this research show the influence of carbapenemase gene levels distributed in both sides of the shaft (0.98), not more than 95% confidence interval (0.92 and 1.05), suggesting that there is no single study overall consolidation effect. In Egger's inspection, when *p* > ∣0.1∣, there is no publication bias. The results of this study display that the overall *p* = 0.728, which suggests a mild publication bias and is accepted in corresponding studies.

This research also has some limitations. As mentioned above, due to the strong randomness, only the data of detecting the “big five” family of carbapenemase genes were included, without the data of other families and the double-detection types. In another respect, because of the imperfections of the corresponding database, the instrument models employed in the studies included in this research could not be analyzed, which is also a defect of our research.

In summary, Xpert Carba-R assay presents a high accuracy in the determination of the “big five” carbapenemase gene families in GNB, together with other merits of simple operation, low price, and less turnaround time. It is a good epidemiological tool for early clinical diagnosis and the prevention of the abuse of antibiotics. With the development of the follow-up versions, Carba-R assay may be an increasingly important diagnostic tool.

## 5. Conclusions

This project carried out an investigation of the accuracy of Xpert Carba-R assay in detecting the “big five” carbapenemase gene families in GNB and came to the conclusion that it could work as a new clinical diagnostic tool and the gold standard. This method has been proved reliable and efficient to detect five important carbapenemase gene families of OXA-48, IMP, NDM, KPC, and VIM. It can be seen that Xpert Carba-R method is an effective tool for early clinical detection, which is suitable for the detection of carbapenase gene in GNB.

## Figures and Tables

**Table 1:** Literature inclusion and exclusion criteria.

Inclusion criteria(according to the “PICOS” standard)	Exclusion criteria
(1) Research object: Gram-negative bacteria (2) Research type: accuracy test, to extract data for identifying carbapenemase genes, only in the English language (3) Measurement indicators: detection rate (4) Diagnostic experimental methods: Xpert Carba-R assay was used to detect carbapenemase genes in GNB	(1) Duplicate literature (2) Abstracts, lectures, reviews, and summaries

**Table 2:** The identification accuracy rate of species from included articles.

Author	Year	Genotype	Total	Events	Detection rate
Tato [9]	2016	OXA-48	40	38	95%
	2016	NDM	26	26	100%
	2016	VIM	31	29	93.55%
	2016	KPC	30	29	96.67%
	2016	IMP	27	26	96.30%

McMullen [10]	2017	OXA-48	12	12	100%
	2017	NDM	27	27	100%
	2017	VIM	7	7	100%
	2017	KPC	84	84	100%
	2017	IMP	1	1	100%

Moore [11]	2017	OXA-48	113	111	98.23%
	2017	NDM	88	88	100%
	2017	VIM	92	87	94.57%
	2017	KPC	110	110	100%
	2017	IMP	80	76	95%

Findlay [14]	2015	OXA-48	100	100	100%
	2015	NDM	100	100	100%
	2015	VIM	100	100	100%
	2015	KPC	100	100	100%
	2015	IMP	24	17	71%

Hoyos-Mallecot [15]	2017	OXA-48	10	10	100%
	2017	KPC	1	1	100%

Kost [16]	2017	OXA-48	9	8	88.90%
	2017	NDM	25	25	100%
	2017	VIM	3	3	100%
	2017	KPC	43	41	95.30%
	2017	IMP	2	2	100%

Vanstone [17]	2018	OXA-48	24	24	100%
	2018	NDM	22	22	100%
	2018	VIM	19	19	100%
	2018	KPC	3	3	100%
	2018	IMP	1	1	100%

Traczewski [18]	2018	OXA-48	89	89	100%
	2018	NDM	78	78	100%
	2018	VIM	82	82	100%
	2018	KPC	84	84	100%
	2018	IMP	40	40	100%

Cointe [19]	2019	OXA-48	10	10	100%
	2019	NDM	11	11	100%
	2019	VIM	7	7	100%
	2019	KPC	10	10	100%
	2019	IMP	1	1	100%

**Table 3:** Characteristics of included articles.

Arthur	Year	Experiment design	Golden standard	Geographical distribution of strains	Source of samples	Total sample	Genotype	Total	Events	Detection rate
Tato [9]	2016	prospective	culture and sequence	UK	383 clinical isolates and 250 contrived isolates	633	OXA-48	40	38	95%
							NDM	26	26	100%
							VIM	31	29	93.55%
							KPC	30	29	96.67%
							IMP	27	26	96.30%

McMullen [10]	2017	retrospective	laboratory-developed PCR assays or must have been previously characterized as part of the CDC	USA, UK and Spain	189 clinical isolates	189	OXA-48	12	12	100%
							NDM	27	27	100%
							VIM	7	7	100%
							KPC	84	84	100%
							IMP	1	1	100%

Moore [11]	2017	prospective	culture and sequence	France	755 clinical isolates and 432 contrived isolates	1187	OXA-48	113	111	98.23%
							NDM	88	88	100%
							VIM	92	87	94.57%
							KPC	110	110	100%
							IMP	80	76	95%

Findlay [14]	2015	retrospective	in-house PCR	UK	450 isolates cultured from 2808C freezer storage or from the sender's original slopes on MacConkey agar plates with a 10 mg ertapenem disc	450	OXA-48	100	100	100%
							NDM	100	100	100%
							VIM	100	100	100%
							KPC	100	100	100%
							IMP	24	17	71%

Hoyos-Mallecot [15]	2017	retrospective	culture and sequence	USA and Italy	241 clinical isolates	241	KPC	1	1	100.00%
							OXA-48	10	10	100%

Kost [16]	2017	retrospective	determined by PCR or whole genome sequencing	USA and Europe	96 clinical isolates	96	OXA-48	9	8	88.90%
							NDM	25	25	100%
							VIM	3	3	100%
							KPC	43	41	95.30%
							IMP	2	2	100%

Vanstone [17]	2018	retrospective	in-house antimicrobial susceptibility testing(AST)	USA	26 clinical isolates and 69 screening samples	95	OXA-48	24	24	100%
							NDM	22	22	100%
							VIM	19	19	100%
							KPC	3	3	100%
							IMP	1	1	100%

Traczewski [18]	2018	retrospective	culture and sequence	USA	428 clinical isolates and 57 fresh isolates	485	OXA-48	89	89	100%
							NDM	78	78	100%
							VIM	82	82	100%
							KPC	84	84	100%
							IMP	40	40	100%

Cointe [19]	2019	prospective	PCR	France	53 clinical isolates	53	OXA-48	10	10	100%
							NDM	11	11	100%
							VIM	7	7	100%
							KPC	10	10	100%
							IMP	1	1	100%

**Table 4:** The quality evaluation results for each study included in the meta-analysis.

Author	Year	QUDAS-2										
		1	2	3	4	5	6	7	8	9	10	11
Tato [9]	2016	Y	Y	UC	UC	UC	Y	UC	UC	Y	Y	Y
McMullen [10]	2017	N	N	Y	N	UC	Y	Y	Y	Y	N	Y
Moore [11]	2017	N	Y	Y	UC	UC	Y	UC	UC	N	Y	N
Findlay [14]	2015	Y	Y	UC	N	UC	Y	Y	Y	Y	Y	N
Hoyos-Mallecot [15]	2017	Y	UC	N	UC	UC	Y	UC	Y	Y	Y	Y
Kost [16]	2017	UC	N	N	N	UC	Y	Y	Y	Y	N	N
Vanstone [17]	2018	Y	Y	UC	N	UC	Y	Y	Y	Y	Y	N
Traczewski [18]	2018	Y	Y	UC	N	UC	Y	Y	Y	Y	Y	N
Cointe [19]	2019	N	N	UC	N	UC	Y	Y	Y	Y	Y	Y

## Data Availability

The data in this study are obtained from the cited literature and have been noted in the manuscript.

## Supplementary Materials

Supplementary Materials S1 Flow diagram of study identification and inclusion.
