# Supplementary material for: Evaluation of Xpert Carba-R Assay for the Detection of Carbapenemase Genes in Gram-Negative Bacteria
Source: Biomed Res Int. 2021 Apr 8;2021:6614812. doi: 10.1155/2021/6614812 (PMC8049809; doi:10.1155/2021/6614812)
Supplement: Supplementary Materials — S1 Flow diagram of study identification and inclusion. [file 6614812.f1.pdf]

PRISMA 2009 Flow Diagram

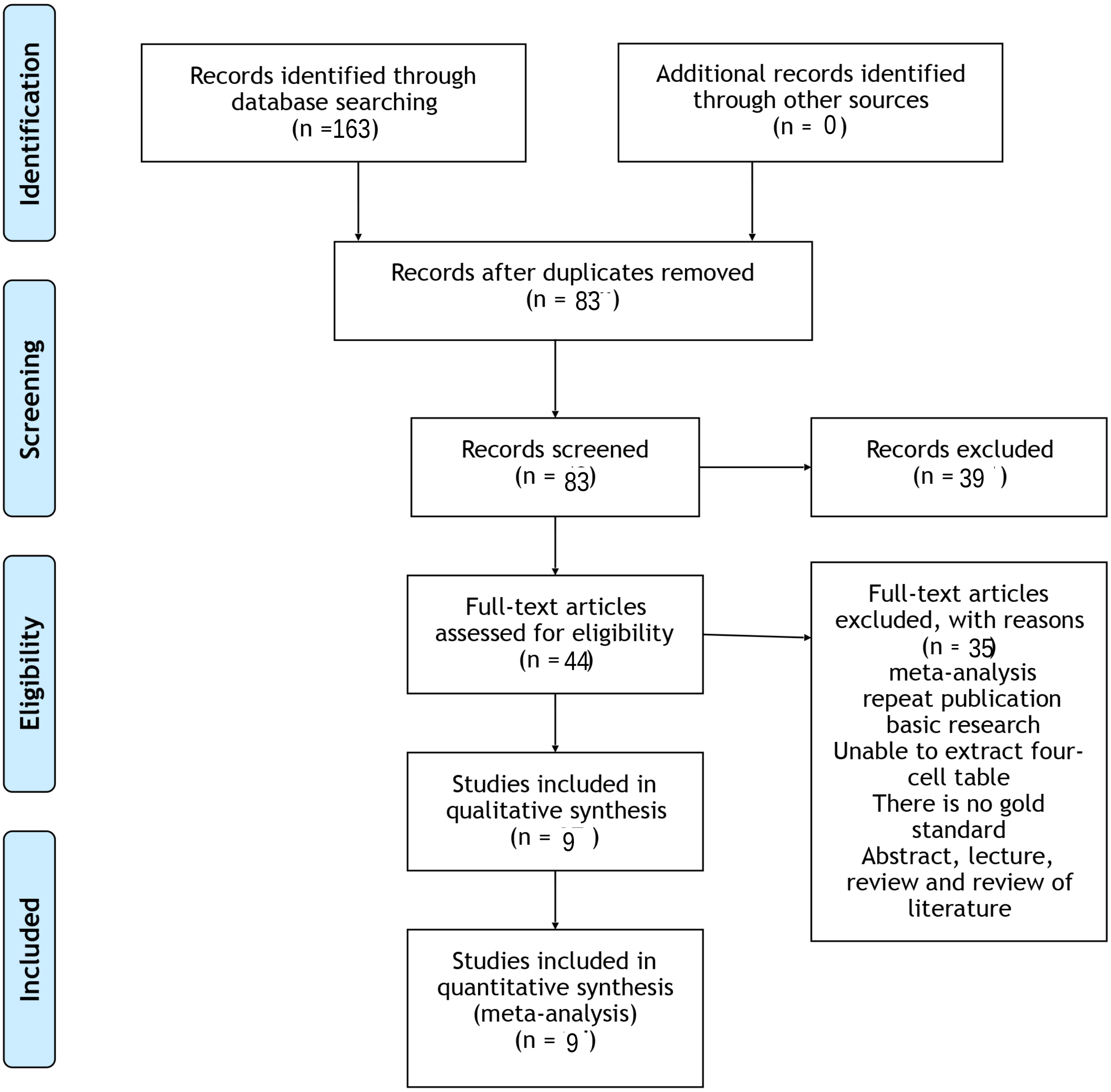

From: Moher D, Liberati A, Tetzlaff J, Altman DG, The PRISMA Group (2009). *P*referred *R*eporting *I*tems for *S*ystematic Reviews and *M*eta-*A*nalyses: The PRISMA Statement. PLoS Med 6(6): e1000097. doi:10.1371/journal.pmed1000097

For more information, visit www.prisma-statement.org.
